# Smart Approach for the Design of Highly Selective Aptamer-Based Biosensors

**DOI:** 10.3390/bios12080574

**Published:** 2022-07-27

**Authors:** Ali Douaki, Denis Garoli, A. K. M. Sarwar Inam, Martina Aurora Costa Angeli, Giuseppe Cantarella, Walter Rocchia, Jiahai Wang, Luisa Petti, Paolo Lugli

**Affiliations:** 1 Faculty of Science and Technology, Libera Università di Bolzano, Piazza Università 1, 39100 Bolzano, Italy; akminam@unibz.it (A.K.M.S.I.); martinaaurora.costaangeli@unibz.it (M.A.C.A.); giuseppe.cantarella@unibz.it (G.C.); luisa.petti@unibz.it (L.P.); 2 Istituto Italiano di Tecnologia, Via Morego, 30, 16163 Genova, Italy; denis.garoli@iit.it; 3 CONCEPT Lab, Istituto Italiano di Tecnologia, Via Enrico Melen 83, 16152 Genova, Italy; walter.rocchia@iit.it; 4 School of Mechanical and Electrical Engineering, School of Chemistry and Chemical Engineering, Guangzhou University, Guangzhou 510006, China; jiahaiwang@gzhu.edu.cn

**Keywords:** aptamer, aptasensor, biosensor, machine learning, molecular dynamic simulation, in silico design

## Abstract

Aptamers are chemically synthesized single-stranded DNA or RNA oligonucleotides widely used nowadays in sensors and nanoscale devices as highly sensitive biorecognition elements. With proper design, aptamers are able to bind to a specific target molecule with high selectivity. To date, the systematic evolution of ligands by exponential enrichment (SELEX) process is employed to isolate aptamers. Nevertheless, this method requires complex and time-consuming procedures. In silico methods comprising machine learning models have been recently proposed to reduce the time and cost of aptamer design. In this work, we present a new in silico approach allowing the generation of highly sensitive and selective RNA aptamers towards a specific target, here represented by ammonium dissolved in water. By using machine learning and bioinformatics tools, a rational design of aptamers is demonstrated. This “smart” SELEX method is experimentally proved by choosing the best five aptamer candidates obtained from the design process and applying them as functional elements in an electrochemical sensor to detect, as the target molecule, ammonium at different concentrations. We observed that the use of five different aptamers leads to a significant difference in the sensor’s response. This can be explained by considering the aptamers’ conformational change due to their interaction with the target molecule. We studied these conformational changes using a molecular dynamics simulation and suggested a possible explanation of the experimental observations. Finally, electrochemical measurements exposing the same sensors to different molecules were used to confirm the high selectivity of the designed aptamers. The proposed in silico SELEX approach can potentially reduce the cost and the time needed to identify the aptamers and potentially be applied to any target molecule.

## 1. Introduction

The development of novel binders for specific targets (e.g., viruses, toxins, pathogens, proteins, cell receptors linked to cancer, etc.) is a continuously growing research area [1] with the goal to simplify the diagnosis and treatment of diseases, detection of contaminants and toxins, as well as quality control [2,3,4,5,6,7,8,9]. Moreover, during the last two years, the ongoing COVID-19 pandemic has underlined the importance of rapid and reliable methods for the large screening of public health [10,11,12,13,14]. Traditional methods to perform such screenings are mainly based on antibodies, although their use presents major drawbacks such as high production time and cost, bad thermal stability, etc. Alternative tools have been proposed [15], in particular, nucleic acid (NA) aptamers have demonstrated selective binding properties toward a broad spectrum of ligands thanks to their three-dimensional (3D) structure. Aptamers derive their name from Latin “Aptus” meaning “to fit” and Greek “meros”, meaning “region” [16]. They are chemically synthesized short single-stranded deoxyribonucleic acids (DNA) or ribonucleic acids (RNA) that serve as selective biorecognition elements [17]. The specific sequence of the aptamers gives them a 3D folding shape that allows them to bind to their targets with high selectivity [18,19,20,21,22,23]. With respect to antibodies, aptamers are cost-effective, easier to synthesize, thermally stable, and simpler to use. Of note, aptamers are now finding more and more applications in nanoscale devices for therapeutic [24,25,26] and sensing applications [27,28,29,30,31]. In particular, the combination of nanopores and aptamers has been demonstrated to be an interesting method for the development of the next generation of highly selective and multiplexed single-molecule sensing devices [28,29,32,33,34,35].

Aptamers are typically selected in vitro, starting with a random pool of RNA or DNA molecules using a process called the systematic evolution of ligands by exponential enrichment (SELEX) [36,37]. The SELEX process consists of multiple cycles of selection and amplification, typically requiring up to 15 rounds and taking a few days to months to be completed [38]. Even if, from their introduction in 1990 [16], the interest in scientific research linked to aptamers has continuously grown, to date, the SELEX method still has some major drawbacks: (i) the maximum theoretical number of sequences in the initial library is limited at 10^15^, thus, it does not enumerate every possible sequence [37,39,40]; (ii) the SELEX may be biased towards certain sequences even though they might present a weak-binding aptamer [39,41,42,43]; and (iii) the presence of an immobilization matrix to which the target is immobilized during SELEX may interact with the NA sequences and give a false-positive result [39,44]. This is why procedures to reduce the number of rounds have been extensively studied. In particular, the use of next-generation sequencing (NGS) and statistical analysis at the end of each cycle have been proposed. Unfortunately, these methods present the drawbacks of increasing the process costs and complexity [37,45,46]. Machine learning models have been recently proposed as an additional tool for aptamer design, but up to now, only a few examples of aptamer-protein analyses have been reported [47,48,49]. To the best of our knowledge, no machine learning models for small aptamer molecules have been reported and they can have a significant impact on several applications such as DNA, toxin, heavy metal, antibiotic, ion, molecular marker, and virus detection.

In recent years, several papers have reported methods to complement the voluminous SELEX technique [45,50,51]. In particular, in silico design and development of aptamers have been proposed to enable the identification of high-affinity aptamers mainly using 3D structural modeling via computer simulations [52]. Different bioinformatics techniques such as docking programs and molecular dynamics simulation (MDS) can be used to study the effect of sequence and structure on function in aptamer design to improve binding affinities [53,54,55,56]. Although these approaches still require a known sequence of aptamer [50,53,57] or performing the traditional SELEX process for at least some rounds followed by NGS [58,59], they may hold the keys to overcoming the drawbacks of the traditional SELEX process in terms of time, cost, and feasibility. Hence, isolating aptamers for a specific target by employing bioinformatics, machine learning, and a rational design represents a challenge with huge potential.

Here, we investigated an extended in silico approach to perform a rational (“smart”) design of aptamers for a test target, represented in this work by ammonium (NH_4_^+^) dissolved in water. We developed, by means of bioinformatics tools and machine learning, a deep learning model able to learn the complex features of an aptamer-target system starting from training data obtained from previously isolated sequences (through standard SELEX). Moreover, we applied molecular docking to the obtained aptamer candidates considering both positive (in this work, NH_4_^+^) and negative targets (trimethylammonia—TMA and dimethylammonia—DMA; two example molecules that can be present with NH_4_^+^ in the food spoilage process). This step of analysis enables only the selection of sequences with a high binding affinity towards the positive target and low binding affinity towards the negative ones. Finally, in order to experimentally prove the performance of the investigated Smart-SELEX method, we selected the top five aptamer candidates obtained from our design procedure, and we used them as recognition elements in a simple electrochemical sensor to detect NH_4_^+^ in water. Electrochemical sensors are extensively used in the field of biosensing due to the low cost of fabrication and their rapid detection time [60,61,62,63]. The use of aptamers as a biorecognition element in electrochemical biosensors (aptasensor) has been previously reported [64,65,66,67]. Here, we explored its use for the detection of ammonia dissolved in water as an ammonium ion at different concentrations. Ammonia is a water-soluble gas that, in a specific range of pH values, dissociates to NH_4_^+^ and OH^−^ (for example, 0.2 ppm of gaseous NH_3_ yields a 2.8 mM ammonia solution [68] which contains 0.2 mM of solvated NH_3_ and 2.6 mM of NH_4_^+^). Consequently, monitoring the ammonium concentration in water could be an indirect measurement of ammonia gas, a well-known toxic gas related to several processes such as food spoilage [68]. We observed that the use of different aptamer sequences in similar electrochemical sensors leads to different responses in terms of electrochemical impedance vs. ammonium concentration. This behavior can be explained by investigating the specific aptamer conformational changes in response to NH_4_^+^. In particular, we used molecular dynamic simulations to correlate the experimental data with the conformational change of the aptamers. Moreover, the high selectivity of the proposed sequences to the positive target with respect to a set of negative experimental targets (in particular, TMA and DMA) has been verified, demonstrating the robustness of the used design. The Smart-SELEX method here proposed can be, in principle, applied to any target molecule, extending the number of negative targets in order to obtain a high selectivity toward multiple analytes. This method can help to accelerate and reduce the cost of aptamer selection, moreover, it can improve the development of diagnostic tool kits and point-of-care devices in terms of cost, time, and precision.

## 2. Materials and Methods

### 2.1. The Smart-SELEX Approach

**Figure 1:**
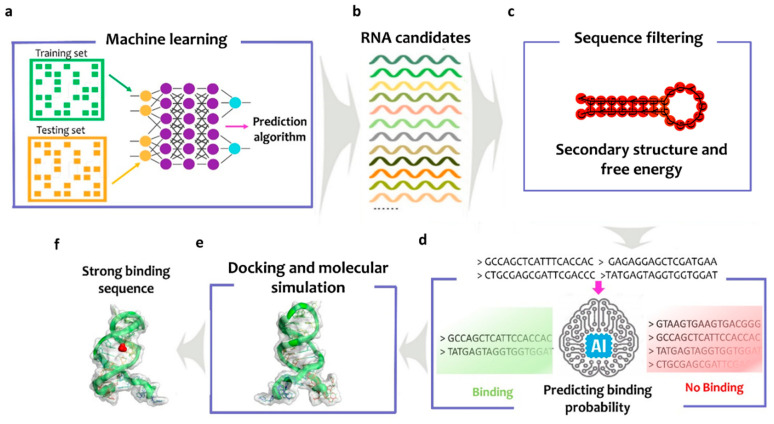
Predicting aptamer sequence using Smart-SELEX: (**a**) training the machine learning model using data collected from the literature data, (**b**) generating the initial RNAs library (10^8^), (**c**) filtering the sequences by calculating the free energy and increasing the number of the loops, (**d**) using the machine learning model developed to predict the binding probability of each sequence with the analyte and ranking them from high to low, (**e**) docking the sequences with the positive and negative analytes and calculating the binding energy, and (**f**) the final aptamer with high affinity towards the target.

Figure 1 displays a schematic diagram of the proposed in silico Smart-SELEX pipeline. Machine learning, docking, and molecular simulations were used to predict the aptamer sequence towards the specific targets (the workflow is described in (Supporting Note #1 and Schematic S1). In particular, a Deep Neural Network (DNN) algorithm was trained using a data set of aptamer sequences selected from the literature considering only aptamers isolated for small target molecules (molecular weight < 900.0 g/mol) such as these reported aptamers [69,70,71,72] (Figure 1a). The size of the data set was 1456 (621 positives and 835 negatives) (Supporting Note #2 explains how the data was selected). Then, a random library (candidate list) of 10^8^ RNA sequences (each 27 nucleotides long) was generated and the repeated RNA sequences were deleted to ensure that all the RNAs of this library are unique (Figure 1b) (Supporting Note #3). Afterward, the candidate list was filtered based on two conditions: (i) the free energy of the self-hybridized structures of the RNA aptamer sequences being higher than −5 Kcal/mol and (ii) the RNA being folded with a maximum number of 20 not bounded nucleotides [73] (Figure 1c) (Supporting Note #4). Then, the trained DNN model was used to predict the binding state (yes or no) between each candidate in the filtered random library and the target (NH_4_^+^) (Figure 1b). Finally, in order to obtain high selectivity towards the target molecule, the free binding energy between the obtained sequences and the positive target is calculated and compared to the same values obtained considering two negative target molecules (such as TMA and DMA). The sequences with a high binding affinity towards NH_4_^+^ and low binding towards the negative targets were kept (Figure 1a).

### 2.2. Machine Learning Model

#### 2.2.1. Data Processing

In our model, aptamer sequences were encoded using the one-hot encoding scheme by assigning the codes {1000}, {0100}, {0010}, and {0001} to the nucleotides A, C, G, and U, respectively. Then, the length of the input aptamers was set for all the data set at 100 base which corresponds to the length of the longest aptamer extracted from the literature. For the aptamers in the data set with a length below 82 bases, we modified the sequence by adding null labels ($) in order to reach the desired length. For the target’s chemical structure representation, the Molecular ACCess System (MACCS) fingerprint was used to convert the molecular structure of the target to a binary vector that can be used as an input for training the machine learning model. The MACCS fingerprint analyzes molecules as a graph, thus, it gives information on the 2D-structural properties of the molecules. RDKit V2 (Open-Source Cheminformatics Software, Landrum, G. 2010, www.rdkit.org/) was used to extract the MACCS fingerprints from the raw SMILES of the targets which represented each target molecule as a length of 166 binary vectors [74]. Then, a correlation matrix was obtained considering the specific aptamer sequence for a target, both as binary vectors. The binding affinity between the set of aptamer sequences and target molecules has been defined with an additional label as “1” or “0” as a function of the reported affinity from the literature (i.e., if a paper reports a specific sequence for a target molecule, we assign 1 to the combination; in contrast, if the aptamer was demonstrated not to be able to bind to the molecule we assign 0). Consequently, the machine learning process took into consideration both positive and negative training data. This enabled us to get a procedure for aptamer-target binding prediction. Finally, the data set was split into a training set and test set, 80% and 20%, respectively.

#### 2.2.2. Model Architecture—Machine Learning

The model learns how a specific sequence can have more or less affinity towards a specific family of targets and consequently enable us to predict affinity towards new targets as in our investigated case (i.e., small molecules). In this approach, a machine learning model was adopted to reduce the number of RNA candidates by predicting the binding probability between the analyte and each RNA candidate. The molecular weight of the small molecules considered here is <900.0 g/mol. These molecules include heavy metals, antibiotics, toxins, ions, drugs, and molecular markers. The model has been treated as a regression problem with the aim of predicting the binding or non-binding state starting from the data obtained from the literature with a decision threshold of 0.5. The Convolutional Neural Network (CNN) was used as a prediction model [75]. The convolutional layers were obtained from the matrix of generated sequences and binary vectors representing the selected small target molecules. The output of the convolutional layers was used as input of the pooling layer with the aim of down-sampling the features learned by the filters [76]. The output of the convolutional and pooling layers was fed to the Fully Connected (FC) layers [77,78]. The CNN model was chosen due to the powerful ability of the filters to extract the local dependencies in the inputs. Therefore, the number and size of the filters affect positively the recognition patterns of the model (Supporting Note #5 and Figure S2) [79,80].

**Figure 2:**
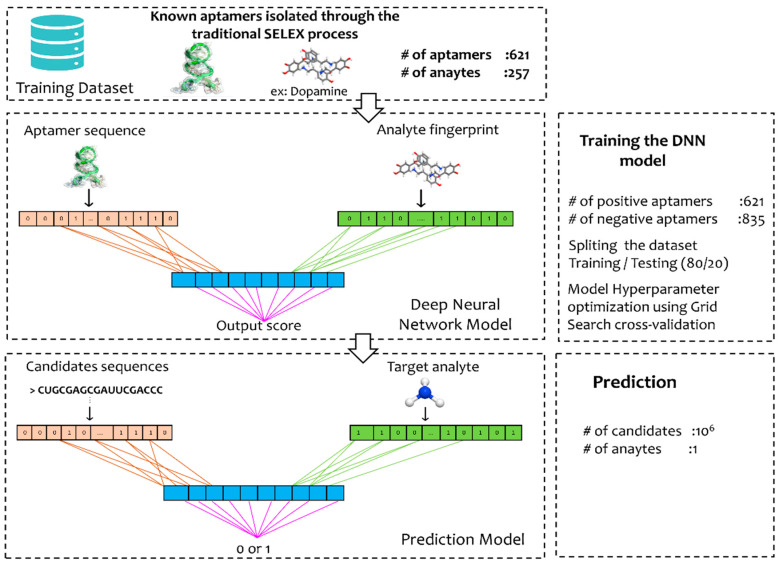
Deep Smart-SELEX model with CNN blocks to learn from aptamer sequences targets MACCS fingerprints and physicochemical properties.

The CNN prediction model adopted in our approach consisted of two separated CNN blocks, one representing the aptamer sequences and the other representing the target molecule fingerprints. This enabled us to learn representations of target molecules’ “MACCS fingerprints” (that represent the physicochemical properties) and how they relate to the aptamers’ sequences. Each CNN block was composed of three one-dimension-convolutional layers in series with several filters of 32, 64, and 96, respectively [81], which were followed by the max-pooling layer. The extracted features were then concatenated and used as inputs to the three FC layers block, where the first two FC layers have 1050 nodes. To avoid the model over-fitting, the first two layers were followed by a dropout layer (regularization technique) of rate 0.1 that set the activation of some of the neurons to 0 [82]. Finally, the last layer of 512 nodes was fed into the output layer (Figure 2 and Figure S2).

#### 2.2.3. Optimization of Hyperparameters

In the adopted deep learning model, different hyperparameters (shown in Table S3), such as the number of filters, length of the filter size, the learning rate, dropout ratio, and aptamer windows, were tuned by employing grid search five-fold cross-validation. According to [83], to check the performance of the developed model, an independent dataset and the following metrics were used: accuracy (Acc.), sensitivity (Sen.), precision (Pre.), and specificity (Spe.):

$$\mathrm{Acc}=\frac{TP+TN}{TP+TN+FP+FN}$$

$$\mathrm{Sen}=\frac{TP}{TP+FN}$$

$$\mathrm{Spe}=\frac{TN}{TN+FP}$$

$$\mathrm{Pre}=\frac{TP}{TP+FP}$$

where *TN* is true negative, *TP* is true positive, *FP* is false positive, and *FN* is false negative. 

### 2.3. Preparing the Library File

Once the machine learning model was defined, the random (10^8^) RNA sequence library was generated by means of an R script. Each candidate in the library has a length of 27 nucleotides. The choice for this length (27 nucleotides, i.e., about 6 nm) is related to the limited sensing distance typically presented in electrochemical sensors (Debye length) which will be used to experimentally prove the proposed method [72,84]. The Vienna RNA package [85] was then used to evaluate the free energy of the secondary structure of the proposed candidates. The free energy and the number of free nucleotides in the sequence (not hybridized) were used to filter the candidates considering the following conditions: free energy lower than −5 Kcal/mol and a maximum number of 20 nucleotides in a single strand configuration (Supporting Note #4. Smart-SELEX for sequence design).

### 2.4. Docking

Docking is the final step in the proposed procedure of “smart”-SELEX. Once the set of filtered RNA aptamer sequences was obtained from the previous steps, we made them interact with the target molecule, i.e., NH_4_^+^. To do so we used the Autodock Tools (ADT) and AutoDock Vina program [86]. Before starting the docking process, polar hydrogen atoms were added to the target and the aptamer molecules. Then the Gasteiger charges were added, and the targets and the RNA aptamer files were converted into pdbqt format using Chimera 1.14. The grid box coordination was (1, 50, and 10) with 128, 50, and 66 points in X, Y, and Z directions, respectively [87]. Moreover, to overcome the receptor flexibility, the binding pocket was selected as a flexible part.

To reduce the docking time, a Message Passing Interface (MPI) scheme was implemented [88]. After 10 runs of docking between the target and the aptamer, an average was taken [55]. Finally, the candidates were ranked based on their binding free energies. A large number of candidates (1896 candidates) were obtained from this procedure. Ranking them by the function of the binding affinity towards the specific target investigated (here NH_4_^+^) and the negative targets (DMA and TMA) enables us to perform proof-of-concept sensing experiments on a small number of candidates. In particular, we selected the first five ranked sequences (other potential sequences obtained from the Smart-SELEX procedure are reported in Supporting Note #6).

### 2.5. Materials and Reagents

Ammonium hydroxide, dimethylamine, trimethylamine, methanol, ethanol, propan-2-ol (IPA), N(3-dimethylaminopropyl)N ethylcarbodiimide (EDC), 11-mercaptoundecanoic acid (11-MUA), N-Hydroxysulfosuccinimide sodium salt (NHS), potassium chloride (KCl), potassium ferricyanide II trihydrate (K_4_[(Fe(CN)_6_] × 3H_2_O), and potassium ferricyanide III (K_3_[(Fe(CN)_6_] were obtained from Sigma Aldrich (Munich, Germany). All chemicals used in this work are analytical grade and were used without any further purifications. The aptamers with the amine group at the 5′-end were ordered from Biomers (Ulm, Germany). Ink pastes, silver chloride ECI 1011, and silver ECI 6038E were purchased from LOCTITE E&C (Bay Point, CA, USA). The polyethylene terephthalate (PET) flexible substrate with a 125-micron thickness was purchased from Mylar (Chester, VA, USA).

### 2.6. Fabrication of the Aptasensor

**Figure 3:**
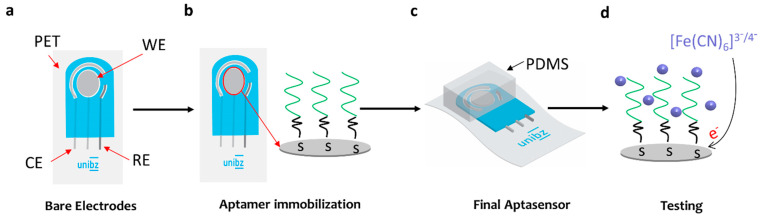
Fabrication flow of aptasensor. (**a**) Screen printing of electrodes, (**b**) immobilization of the aptamers using a self-assembled-monolayer, (**c**) final aptasensor cover by a PDMS chamber, and (**d**) testing of the performance of the aptasensor by using [Fe (CN)_6_]^3−/4−^ as an electrolyte.

The top five aptamers that resulted from the Smart-SELEX approach were purchased and used as biorecognition elements in the aptasensor for ammonium detection. The sensors were fabricated by screen-printing (semi-automatic screen-printing machine -Aurel C920-, Milan, Italy) the electrodes on a flexible polyethylene terephthalate (PET) substrate. Figure 3a shows a schematic of a screen-printed flexible electrode, consisting of a silver (Ag) Counter Electrode (CE), an Ag-working electrode (WE), and an AgCl reference electrode (RE), with a total length of 22 mm and a width of 8 mm. The sensor was fabricated as follows: firstly, CE, WE, and the lower half part of the RE were screen-printed and cured at 120 °C for 15 min, afterward, the upper half of the RE was screen-printed using the AgCl ink. Finally, the electrodes were passivated with a screen-printed dielectric layer in order to contain the electrolyte droplet to ensure a reproducible working area size. After that, the electrodes were ultrasonically cleaned in IPA and then ultrapure water for 3 min 5 min, respectively. After that, the Ag working electrodes were immersed in 1 mM 11-MUA for 24 h to form the SAM-COOH layer. The 11-MUA has a thiol group on one side and a carboxylic group on the other side, where the terminal -SH group of 11-MUA was attached to the Ag electrode, leaving a free carboxylic group to be attached covalently with the amine group at −5′ end of the aptamers. Furthermore, 8 µL of 1 µM aptamers, 300 mM EDC, and 35 mM NHS pH 7.0 were drop-casted on top of the working electrode and air-dried for 2 h.

### 2.7. Electrochemical Measurements

VersaStat 4 potentiostat galvanostat (Princeton applied research, Ametek scientific instruments, Oak Ridge, TN, USA) controlled by VersaStat studio was used for all cyclic voltammetry (CV) sweeps. The working area (CE, WE, and RE) was covered with 50 µL of 1 mM [Fe (CN)_6_]^3−/4−^ containing 0.1 M KCl + 3 mM Mg^2+^ (MgCl_2_) in PBS buffer, while the measurements were performed with a scan rate of 100 mV/s and a scan potential between −0.8 to 0.8 V. Electrochemical impedance spectroscopy (EIS) measurements were performed in a frequency range of 10 mHz–1 MHz, an AC amplitude of 50 mV and a sampling rate of 60 points using [Fe (CN)_6_]^3−/4−^ as an electrolyte. The aptamers affinity values (Kd) values were also calculated by nonlinear regression analysis from the calibration curve (employing the Langmuir–Hill equation).

### 2.8. Chemical Analysis

Infrared spectra were obtained with a Fourier Transform Infrared Spectrometer (Invenio FTIR, Bruker, Billerica, MA, USA) using a diamond crystal. The spectra were recorded in the range of 500–4000 cm^−1^, and a resolution of 4 cm^−1^.

### 2.9. Molecular Dynamics Simulations

The MD simulations of the NH_4_^+^-aptamer interaction were performed in Gromacs using the AMBER99SB-ildn force field. The aptamer was placed in the center of a water box of suitable dimensions according to the size of the different aptamers, then NH_4_^+^ was inserted into the box, with a distance between the box and the surface of the aptamer of 1 Å. Afterward, water molecules of the TIP3P model and Na+ ions for charge neutrality were inserted into the box. AmberTools in Gromacs was used to generate the analytes topology files. By applying the steepest descent algorithm and considering periodic boundary conditions (PBC) in all directions, energy minimization was performed. Then, the systems were equilibrated in NVT (where the constant number (N), volume (V), and temperature (T), respectively) and NPT (constant number (N), pressure (P), and temperature (T), respectively) and assembled with a time step of 2 fs for 100 ps. Finally, the simulation was performed at a pressure and temperature of 1.01 atm and 294 K for 100 ns, respectively. The equilibrated system was simulated in the NPT ensemble for 10 ns 82.

The molecular dynamic simulations were performed with Intel(R) Xeon(R) W-2255 CPU @ 3.70 GHz (Intel Corporation, Santa Clara, CA, USA) and the GPU NVIDIA Quadro RTX 4000 (Nvidia Corporation, Santa Clara, CA, USA) with Ubuntu 18.04. LTS. Gromacs tools were used to analyze the trajectories (e.g., Radius of gyration, RMSD) of the MD simulations.

## 3. Results

### 3.1. Smart-Selex

The machine learning stage employed in this work enabled the improvement of the design of aptamer sequences, reducing the number of candidates, and hence, decreasing the computational time required by docking and molecular dynamic simulations. As previously mentioned, the adopted CNN prediction model consisted of two separated CNN blocks, one representing the aptamer sequences and the other representing the target molecule fingerprints. Figure 2 shows the general workflow of the aptamer sequence and target one-hot encoding, cross-validation, and final training. Grid search cross-validation was used to tune the hyperparameters of ‘DeepSelex’, as follows: first, a grid of hyperparameters was created containing a wide range of values, then five-fold cross-validation was used to evaluate the model performance. The model performance before and after tuning the hyperparameter was evaluated in terms of different metrics. The model before tuning had an Acc:0.804, Spe:0.816, Sen: 0.812, and Pre: 0.808, however, these metrics after tuning were improved with an Acc: 0.835, Spe: 0.829, Sen: 0.845, and Pre: 0.831. The selected values of hyperparameters for DeepSelex are summarized in Table S3. Moreover, by increasing the hidden neuron size to more than three layers we obtained an over-fitting. Hence, the number of layers was set to three with 1024 neurons for the first two layers and 512 for the third layer, respectively. The training performance of this model was measured as categorical cross-entropy loss. After 25 epochs, an average validation loss of 0.71 ± 0.08 was obtained, hence, the number of epochs for training the model was set at 25. The final optimized model with the best performance was saved and used for predicting the binding probability of the RNA candidates towards NH_4_^+^.

Table 1 reports the top five sequences obtained from the proposed Smart-SELEX method with the binding energy towards NH_4_^+^, DMA, and TMA. As discussed in the method section and reported in detail in SI, the procedure comprises three successive steps of computation: (i) initial sequence library selection and filtering; (ii) machine learning-based prediction of binding towards target molecules; and (iii) docking (Figure S3 shows the evolution of the number of RNA candidates). The first step, namely the filtration of candidates based on the free energy of the self-hybridized structures and a maximum number of 20 nucleotides in a single strand configuration, enabled the reduction in the number of RNA sequence candidates from 10^8^ down to 10^6^ potential sequences. Afterward, 10^6^ ± 71,992 candidates (average of three runs) were fed into the machine learning model to predict the binding probability with a threshold of 0.5; this step reduced the number of RNA candidates to 38,327 ± 1099 potential candidates. Finally, positive docking (towards NH_4_^+^) was performed on the 38,327 RNA candidates; 1896 ± 71 RNA candidates passed this step, then negative docking (towards TMA and DMA) was performed on the 1896 RNA candidates. At the end of the process, it was possible to select a few candidates with binding energy towards the target (NH_4_^+^) in the range between −9.85 and −6.6 Kcal/mol. To note, the docking procedure allows for selection among sequences with a good affinity towards NH_4_^+^ and at the same time with a bad affinity towards the other tested targets (not desiderated analytes—TMA and DMA) (shown in Figure S4). Moreover, Supporting Note #7 shows the required time for each step in the Smart-SELEX approach.

### 3.2. Aptasensor Performance

The five aptamers were tested for sensitivity and selectively towards NH_4_^+^ by using a simple electrochemical sensor functionalized with the designed aptamer sequences. A direct comparison between the selected sequences and a random “control” sequence from the initial candidate list was also done by using an additional control sensor.

To monitor the fabrication process of the aptasensor and to confirm the proper immobilization of the aptamers on the surface of the electrode, two electrochemical techniques (EIS and CV) were applied to investigate the change induced by the aptamer’s immobilization on the WE in terms of electrode surface resistance and electron transfer rate. Moreover, FTIR was performed after the addition of 11-MUA and immobilization of the aptamers to monitor the chemical bonding formation. The results of this characterization can be seen in Supporting Note #8.

**Figure 4:**
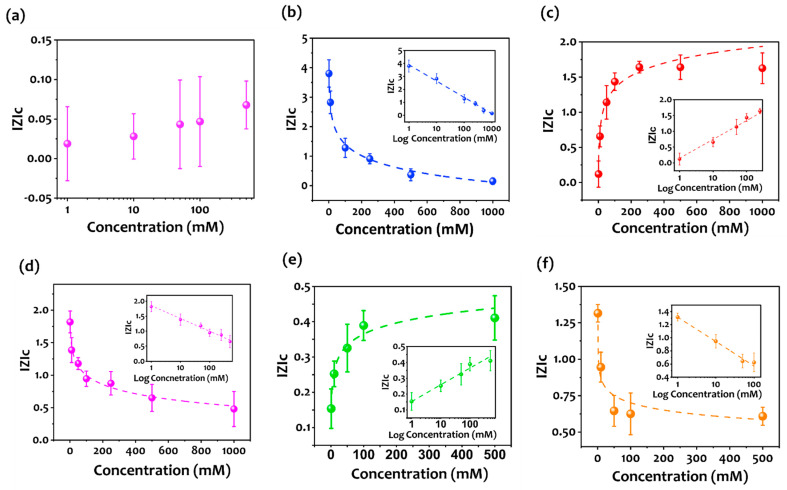
(**a**–**f**) Sensors’ response vs. Log ammonium concentrations for the five aptamers obtained from the Smart-SELEX. Comparison with a Control random sequence. N = 3 samples. (**a**) Control aptamer, (**b**) aptamer 1, (**c**) aptamer 2, (**d**) aptamer 3, (**e**) aptamer 4, and (**f**) aptamer 5.

The sensitivity of the developed aptasensor was evaluated with various concentrations of NH_4_^+^ in water at a pH close to 7. Figure 4 shows the relative impedance modulus |Z|_c_ defined as |Z|_f_ − |Z|_0_/|Z|_0_ of the aptasensor’s responses to different target concentrations (from 1 mM up to 500 mM), where |Z|_0_ is the impedance modulus of the blank solution for each device and |Z|_f_ is the final impedance modulus after 15 min incubation with the desired target concentration taken at the modulus at 5 Hz. To note, at the used pH, 1mM of ammonia dissolved in water can be an indirect measure of ammonium in the gas phase at concentrations below 1 ppm [68]. As can be observed in Figure 4, the impedance modulus change exhibits a good linear correlation with the logarithmic value of the target concentration for the tested aptasensors. The correlated linear equations of the aptasensors are reported in Table 2.

As shown in Figure 4a, the sensor functionalized with an aptamer with a random sequence (control) showed no significant change in the responses of the device towards the target. In contrast, the measurements performed using the five selected sequences (aptamer candidates) presented good linear responses in the explored range of concentrations. It is very important to note that significant differences were obtained from the different sensors. In particular, while the sensors functionalized with Apt1, Apt3, and Apt5 (Figure 4b,d,f) show a decreasing |Z|_c_ as a function of increasing NH_4_^+^ concentration, the sensors that used Apt2 and Apt4 (Figure 4c,e) show the opposite behavior. In order to explain these phenomena, we need to consider the working principle of an aptamer-based electrochemical sensor. As previously demonstrated [89,90], the change in impedance is due to different aptamers’ conformational change (Figure S7). In the presence of the analyte, the aptamer undergoes a conformational change that can extend or shorten its length. Consequently, it can get more distant or closer from the surface of the electrode. Hence, the behaviors observed experimentally can be reasonably due to some different conformational responses of the aptamers used during the interaction with the target molecule. Considering the effect of a negatively charged molecule (aptamer) on the Z value, we hypothesized that Apt1, Apt3, and Apt5 get farther from the working electrode, in contrast, Apt2 and Apt4 get closer when interacting with NH_4_^+^. To confirm this hypothesis, we performed a set of MD simulations and calculated the gyration radius (R_g_) values which give us information on the elongation/conformation change of the aptamers in the absence and presence of the analyte.

### 3.3. Molecular Dynamic Simulation

**Figure 5:**
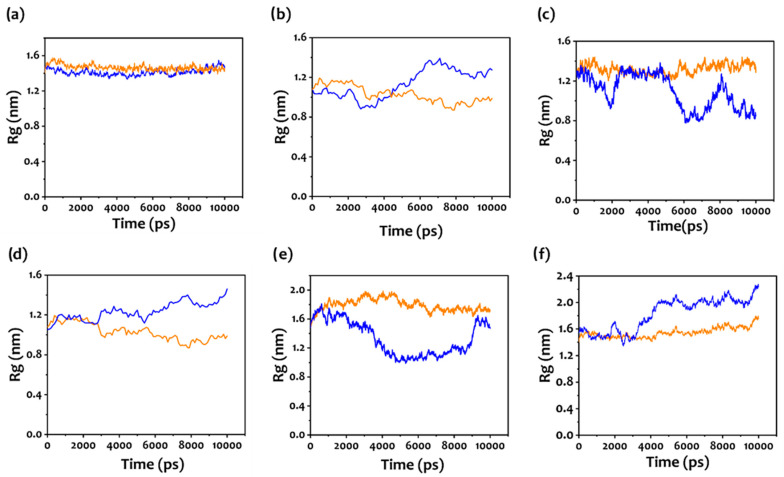
(**a**–**f**) The radius of gyration (R_g_) for the different aptamers with (blue) and without (orange) the target molecule (NH_4_^+^); (**a**) Control aptamer, (**b**) aptamer 1, (**c**) aptamer 2, (**d**) aptamer 3, (**e**) aptamer 4, and (**f**) aptamer 5.

Figure 5 illustrates the gyration radius (R_g_) of the different aptamers as obtained from the molecular dynamic simulations performed for 10 ns. The duration of the MDS was chosen based on the RMSD plot (Figure S8), as can be observed, a simulation of 10 ns was enough for the system to reach a stable state.

As it can be observed, while the aptamers are rather stable in conformation before the interaction with the target molecule, all the designed molecules change their conformation once NH_4_^+^ is included in the simulation. Figure S9 shows the 3D structure of Aptamer1 extracted at the end of the MDS simulation, illustrating the various components of aptamers. According to the diagram, the binding site is located at the stem-loop (or hairpin loop). Therefore, the ammonia molecule was interacting and binding with the stem-loop. The observed conformational changes were in good agreement with the experimental results. Indeed, Apt1, Apt3, and Apt5 (Figure 5b,d,f) showed an increasing value of R_g_ in response to NH_4_^+^, demonstrating a less compact structure in the presence of the target. This is a clear indication of a conformational change and a consequent different distance from the surface of the electrode [72,91,92]. In fact, the curves reported in Figure 4b,d,f for these three aptamers show that the impedance changes decreased when the concentration of ammonium increased. A complementary effect can be observed for Apt2 and Apt4 (Figure 5c,e), in these cases, the R_g_ decreases once NH_4_^+^ interacts with the aptamer sequences. This suggests that the observed results from the experimental electrochemical measurements are related to this different behavior of the functional molecule [92,93]. To note, the random sequence used in our experiment does not change its conformation (Figure 5a) during the interaction with NH_4_^+^, hence confirming the quality of the designed sequences.

Moreover, to better confirm the binding site, the Root Means Square Fluctuation (RMSF) of the best performing aptamer (Aptamer1) in the absence and presence of the analyte was also calculated. RMSF is used to evaluate the flexibility and the fluctuation of atoms. The RMSF was calculated for the 100 ns trajectory of Aptamer1- NH_4_^+^ (Figure S10). The bases (nucleotides) of the Aptamer1 binding site (binding pocket) are C12–C19. The RMSF values for the single bases were decreased in the absence and presence of ammonia which is an indication of a formation of a rigid binding site.

### 3.4. Selectivity

**Figure 6:**
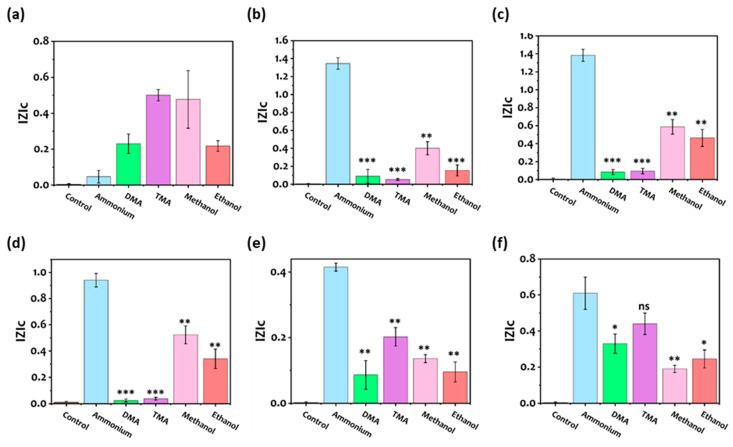
(**a**–**f**) Selectivity test for the detection of ammonium. (**a**) Control aptamer, (**b**) aptamer 1, (**c**) aptamer 2, (**d**) aptamer 3, (**e**) aptamer 4, and (**f**) aptamer 5. The concentrations of ammonium and non-target chemicals were 100 µM. Data are expressed as means ± SE; Statistical significance was assessed with a two-way ANOVA with Dunnett’s multiple comparison test for ammonium and other analytes, * *p* ≤ 0.5, ** *p* ≤ 0.05, *** *p* ≤ 0.0001, ns—not significant.

The final aspect to be considered and verified in our method regards the ability of the proposed sequences to selectively detect the target molecule with respect to other molecules considered during the computation optimization. This is a major aim in the smart design of aptamer sequences for sensing. Indeed, during the computational optimization, interfering molecules were considered, dimethylamine and trimethylamine, in particular, that react with NH_3_ as major contaminants in food spoilage [94,95]. In order to verify that the developed devices are highly selective toward the specific target, experimental tests were performed on the selected aptamers. Figure 6 shows the results, where methanol and ethanol have also been included in the analyses. These two additional molecules are known to be involved during food spoilage processes, similar to NH_3_, TMA, and DMA [96]. As expected, the different aptamers were detected with different sensitivity to the target and non-target analytes (DMA, TMA, methanol, and ethanol). While the non-selectivity to NH_4_^+^ in the case of the control aptamer (Figure 6a) is clear, with the five sequences obtained from our design method the sensitivity towards NH_4_^+^ was always significantly higher with respect to the other analytes. In particular, for Apt1, Apt2, and Apt3 (Figure 6b–d), the |Z|_c_ measured with NH_4_^+^ were about 15 times the values obtained with TMA and DMA. This is not the case for Apt4 and Apt5 (Figure 6e,f) where the performance in terms of selectivity was decreased. Notably, the set of sequences obtained from the Smart-SELEX was ranked in agreement with a binding affinity towards the three analytes, so we can expect that only the top sequences show very good performances (in our case, the top three). In all the cases, the aptamers were only partially selective towards methanol and ethanol. This is partially justified since, during the Smart-SELEX process, methanol and ethanol were not taken into consideration (only TMA and DMA were considered), hence, by including more positive or negative targets the selectivity can be improved and we can also tune the aptamer based on the desired application. Moreover, the aptamers were more sensitive toward methanol than ethanol and this may be due to the high solubility of methanol compared to ethanol and ammonia, with Henry’s law solubility constant of 230 (mol × kg^−1^ bar^−1^), 190 (mol × kg^−1^ bar^−1^), and 59 (mol × kg^−1^ bar^−1^), respectively [97]. Moreover, the low selectivity of Apt4 and Apt5 was further explored. It is hypothesized that Apt4 and Apt5 possessed more than one binding site, allowing the TMA and DMA to bind. Therefore, we examined the secondary structure of the five aptamers (Figure S4) which revealed that Apt1, Apt2, and Apt3 have only one binding site. In contrast, APT4 and APT5 have two binding sites, which might enable TMA and DMA to bind. In order to confirm this, the binding site of TMA on APT4 and APT5 was investigated in terms of MDS. As depicted in Figure S12, TMA is bound to the second pocket rather than the first that ammonIa is bound to. Additionally, the fact that APT4 and APT5 had a lower ranking indicates that the machine learning model learned that the presence of more than one binding site affects the selectivity of an aptamer.

## 4. Conclusions

In summary, this paper demonstrated that an in silico approach for aptamer selection towards small organic molecules targets is a feasible and promising strategy. We named this method Smart-SELEX, as it represents an improvement over the conventional SELEX process and can be completely performed in silico. The computational method illustrated here allowed the design of specific oligo sequences with optimized binding energy towards a set of targets (positive and negative). Although only three molecules were considered during the optimization, namely, NH_4_^+^, DMA, and TMA, the method allows for the inclusion of more positive or negative targets in order to improve the selectivity and tune the aptamer based on the application. As expected, the different aptamers used in our experiments had different responses to the analyte. By using electrochemical measurements, it has been possible to investigate the conformational changes of the used aptamers by combining experimental measurements with molecular dynamic simulations. The selectivity has been verified towards the analytes considered during the optimization of the design. The use of two additional analytes suggested that, in order to obtain high selectivity, more molecules must be considered in the in silico process. Our work shows that the Smart-SELEX approach could hold the keys to overcoming the drawbacks of the traditional SELEX process in terms of time, cost, and the feasibility to isolate aptamers for sensing by employing bioinformatics, machine learning, and a rational design. Future work would be to increase both the number of positive and negative targets and the candidates’ library from 10^8^ to 10^15^ to cover all the possible sequences. These may improve the performance of the selected aptamers to be used in multiple aptasensors in nanoscale devices such as single-molecule detectors and nanopores. Finally, building a pipeline to make the approach automatic will improve the performance in terms of accuracy and speed by reducing human intervention. The proposed method can be, in principle, extended to different families of targets, such as proteins, viruses, etc. As is well known, in the development of a diagnostic tool for a specific target, a biorecognition element (antibody or aptamer) is needed. The isolation of antibodies or aptamers can take days or even months and it is an expensive process. A smart in silico method makes this development less expensive, in fact, the approach proposed in this work has almost zero cost except for the computational power.

## Figures and Tables

**Table 1:** Top five candidates selected by the Smart-SELEX.

Rank	Candidate Sequences	Binding Energy (kcal/mol) NH4^+^ from MDS	Binding Energy (kcal/mol) DMA from MDS	Binding Energy (kcal/mol) TMA from MDS	Kd (mM)	Detection Range (mM)	Limit of Detection (mM)
Aptamer 1	CCAUGUAAGCGCGGUACUCUUACGUGA	−9.85	−3.8	−3.21	36.59	1–1000	0.08
Aptamer 2	UCGCGUCUAGCCCAUUGAUAGGCCCGA	−9.67	−4.46	−3.53	16.11	1–500	0.37
Aptamer 3	UCCACGUGGUGCCAUACUCCGGCGUGG	−9.37	−5.26	−4.21	131	1–1000	0.61
Aptamer 4	CCUCUCAGGCUUGUACUGCCACGAGGA	−8.66	−4.86	−4.78	6,6	1–500	0.40
Aptamer 5	GCCCUGGGCCGCUCAUUCCCUCUGGCU	−8.31	−5.02	−5.43	50	1–500	0.16

**Table 2:** Linear equations of the aptasensors.

Aptamer nr.	|Z|_c_	Paerson’s r
Control (Random sequence)	(−0.01) × logC + 0.01	0.978
Aptamer 1 (Apt1)	(−1.30) × logC + 3.96	0.995
Aptamer 2 (Apt2)	(0.632) × logC + 0.098	0.99
Aptamer 3 (Apt3)	(−0.416) × logC + 1.838	0.987
Aptamer 4 (Apt4)	(0.106) × logC + 0.151	0.983
Aptamer 5 (Apt5)	(−0.26) × logC + 1.27	0.968

## Data Availability

Not applicable.

## Supplementary Materials

Details on the machine learning model, experimental procedure, and aptasensor characterization. The following supporting information can be downloaded at: https://www.mdpi.com/article/10.3390/bios12080574/s1, Supporting Note #1. Smart-SELEX workflow, Schematic S1. Smart-SELEX workflow, Supporting Note #2. Preparing the positive and negative data, Table S1. An example of positive and negative data was collected from the literature, Supporting Note #3. Generation of RNA candidates, Supporting Note #4. Smart-SELEX for sequence design, Table S2. The free energy secondary structure of RNA aptamers binding different ligands, Figure S1. 3D structure of aptamer1 extracted from molecular dynamic simulation, Figure S2. Deep-selex model structure, Table S3. selected values of hyperparameters for DeepSelex, Supporting Note #6. Docking, Table S4. Top 10 candidates sequences, Supporting Note #7. Required time, Figure S3. Evolution of the number of RNA candidates through the Smart-Selex approach, Figure S4. The secondary and tertiary structures of five top aptamers, Supporting Note #8. Sensor characterization, Table S5. The sequences of the aptamers and their modifications, Figure S5. (a) Cyclic voltammogram curves, and (b) EIS spectra of the stepwise modified electrode in 1 mM [Fe (CN)_6_]^3−/4−^ an aqueous solution containing 0.1 M KCl: bare (in blue), (b) bare-aptamer (in orange), Figure S6. IR spectra of the stepwise modified electrode, Figure S7. different conformational changes of the aptamers and the effect on the electron transfer charge (ETR). (A) after the interaction with the analyte the aptamers become less compact and bend far from the surface of the working electrode hence, increasing the ETR. (B) after the interaction with the analyte the aptamers undergo a conformational change and bend closer to the surface of the working electrode hence the ETR decreases, Supporting Note #9. Molecular Dynamics Simulation, Figure S8. RMSD values of aptamer1, aptamer2, and aptamer3, Figure S9. 3D structure of aptamer1 interacted with Ammonia extracted from molecular dynamic simulation, Figure S10. RMSF of aptamer I atoms during 25 ns MD simulation. RMSF comparison between Aptamer (orange line) and Aptamer1 + NH_4_^+^ (blue line), Figure S11. 3D structure of 5 top aptamers interacted with Ammonia extracted from molecular dynamic simulation. (a) aptamer1, (b) aptamer2, (c) aptamer3, (d) aptamer4, (e) aptamer5, Figure S12. 3D structure of (a) aptamer4 and (b) aptamer5 interacted with TMA, Supporting Note #10. Sensors regeneration and stability, Figure S13. The change in Aptasensor 1 response after different cycles of regenerations (detecting 100 mM of ammonia), Figure S14. The stability of aptasensor 1 overtime (detecting 100 mM of ammonia), Supporting Note #11. Detection of real sample, Table S5. NH4+ detection in a real sample.
