# Supplementary material for: Smart Approach for the Design of Highly Selective Aptamer-Based Biosensors
Source: Biosensors (Basel). 2022 Jul 27;12(8):574. doi: 10.3390/bios12080574 (PMC9405846; doi:10.3390/bios12080574)
Supplement: Supplementary file 1 [file biosensors-12-00574-s001.zip › biosensors-1780592-supplementary.pdf]

SUPPORTING NOTE

Smart Approach for the Design of Highly Selective Aptamer-Based Biosensors

Ali Douaki [1,*], Denis Garoli [2], A. K. M. Sarwar Inam [1], Martina Aurora Costa Angeli [1], Giuseppe Cantarella [1], Walter Rocchia [3], Jiahai Wang [4], Luisa Petti [1] and Paolo Lugli [1,*]

[1] Faculty of Science and Technology, Libera Università di Bolzano, Piazza Università 1, 39100 Bolzano, Italy; akminam@unibz.it (A.K.M.S.I.); martinaaurora.costaangeli@unibz.it (M.A.C.A.); giuseppe.cantarella@unibz.it (G.C.); luisa.petti@unibz.it (L.P.)
[2] Istituto Italiano di Tecnologia via Morego, 30, 16163 Genova, Italy; denis.garoli@iit.it
[3] CONCEPT Lab, Istituto Italiano di Tecnologia, Via Enrico Melen 83, 16152 Genova, Italy; walter.rocchia@iit.it
[4] School of Mechanical and Electrical Engineering, School of Chemistry and Chemical Engineering, Guangzhou University, Guangzhou 510006, China; jiahaiwang@gzhu.edu.cn
* Correspondence: aldouaki@unibz.it (A.D.); paolo.lugli@unibz.it(P.L.)

Supporting Note #1. Smart-SELEX workflow

Schematic 1 shows the different steps of the Smart-SELEX. The Smart-SELEX was designed with the same principle as the conventional SELEX, starting with a pool of random RNA sequences, then introducing the target analyte (positive selection) and other analytes that can be found in the same environment (negative docking), and finally, ranking the candidates.

- Training the machine learning model: Prior to performing the Smart-SELEX, a machine learning model was trained to predict the binding probability of any target analyte with any random sequence. To accomplish this, a training dataset was collected and the machine learning algorithm was trained.
- Generating a Random Library: As with conventional SELEX, the Smart-SELEX approach begins with a pool of random sequences. Accordingly, random RNA sequences were generated with the removal of duplicated sequences.
- Filtration: Afterward, random sequences were filtered by employing different criteria (**Supporting Note #4**).
- Machine learning: The sequences that survived the filtration step then were fed into the machine learning model previously trained with the aim of ranking the sequences based on their probability to bind the target analyte (in this case, NH_4^+).
- Positive docking: The binding energy between the sequences derived from the machine learning model and NH4+ was calculated. The sequences with the highest binding energies towards NH4+ were retained.
- Negative docking: The binding energy between the sequences from the positive docking and DMA and TMA (they can be an interferent analyte) were calculated to discard the sequences that had binding energy towards DMA and TMA. This step aimed to improve the selectivity of the aptamers.

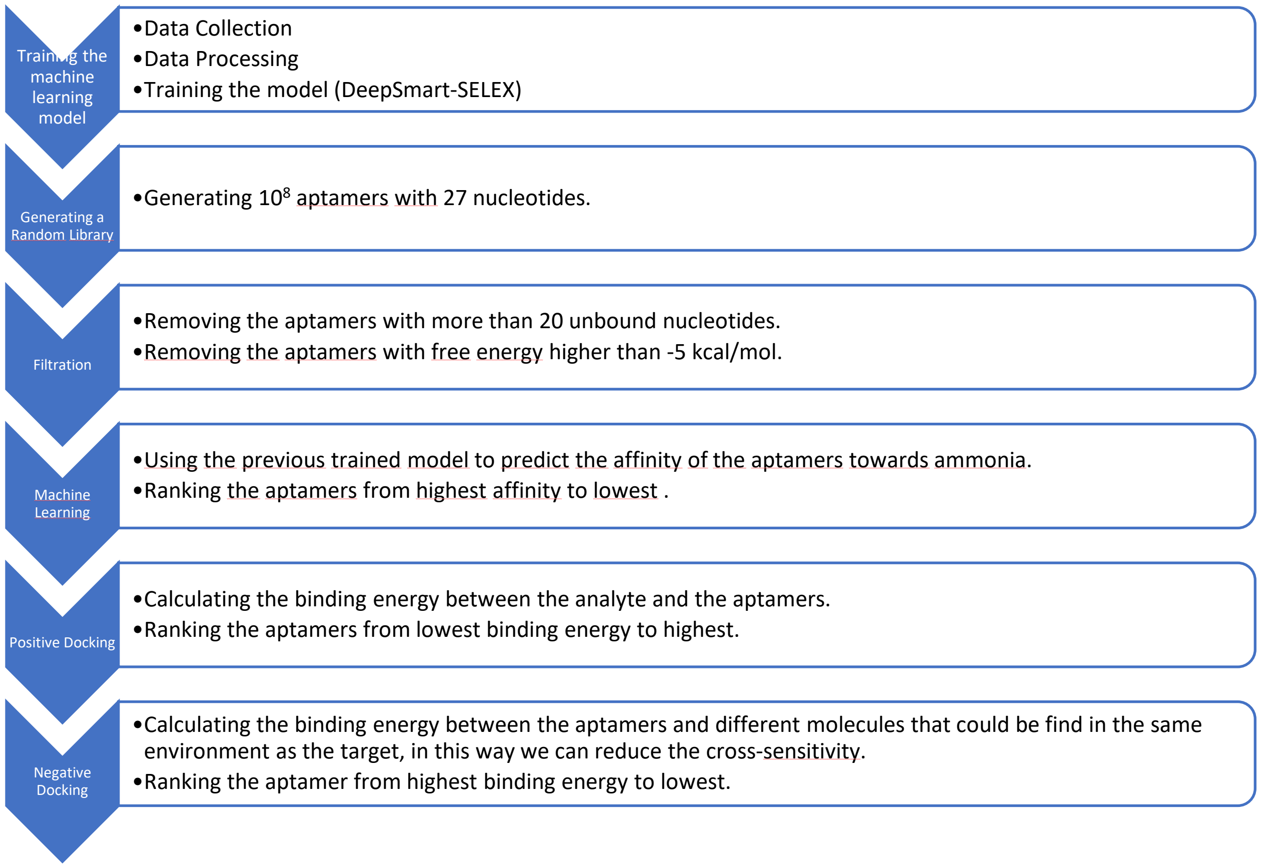

Scheme S1. Smart-SELEX workflow.

Supporting Note #2. Preparing the positive and negative data

The 621 positive datasets were selected from the literature and showed good affinity (kd) from the range of uM to nM towards different targets (dopamine, aflatoxin, etc.) were used as positive data. On the other hand, the negative datasets were selected from the published work where the selectivity of the aptamer was investigated. For instance, in Hongzhi´s work [1] they used an RNA aptamer "GGAUCCCGACUGGCGAGAGCCAGGUAACGAAUGGAUCC" that is sensitive toward Malachite green (MG) and investigated the selectivity towards Chloramphenicol and Leucomalachite green (LMG). In this case, MG was chosen to be in the positive data set and Chloramphenicol and LMG were chosen to be in the negative data set. In nutshell, positive data can be defined as the reported aptamers in the literature that were experimentally proven to bind to a certain target with high affinity. Negative data is defined as the aptamers reported in the literature that have been experimentally proven to not bind to analytes (as shown in the table below). Table S1 shows an example of positive and negative data.

Table S2. An example of positive and negative data collected from the literature [1].

Aptamer sequence	Target	Positive/Negative	Label
GGAUCCCGACUGGCGAGAGCCAGGUAACGAAUGGAUCC	Malachite green	Positive	1
GGAUCCCGACUGGCGAGAGCCAGGUAACGAAUGGAUCC	Chloramphenicol	Negative	0
GGAUCCCGACUGGCGAGAGCCAGGUAACGAAUGGAUCC	Leucomalachite green	Negative	0

Supporting Note #3. Generation of RNA candidates

In this work, RNA candidates were chosen instead of DNA candidates due to the fact that the Smart-SELEX approach reported in this work relies on the accurate prediction of the 3D structure of the aptamer candidate that is used for docking purposes, thereby, an accurate prediction of the 3D structure of the aptamer is imperative. Hence, RNA aptamers were chosen because their 3D structure prediction is more accurate. The initial library consisted of 10^8 random RNA candidates, and the length of the RNA candidates was set to be 27 for two reasons:

- McKeague et al. performed a literature review on the reported aptamers and then analyzed the length of the selected aptamers, observing that the most frequent aptamers have a length in the range of 25-30 nucleotides [2] .

- Nakatsuka et al. reported that in the sensing field (field-effect transistors), the Debye length is a major problem. Where, in the presence of the analyte, the target analyte induces a change in terms of charge generation. If this charge generation is longer than a certain distance from the surface of the electrode, this change induced by the target will not be captured; this distance is called the Debye length. Hence, aptamers gained importance in the sensing field due to their small size compared to the traditional biorecognition elements, such as antibodies and enzymes, and therefore, selecting an aptamer with a short length is required to overcome the Debye length. The Debye length in PBS solution is around 0.7 nm. For instance, in field-effect transistors, to effectively detect the change induced by the analyte during the electrical measurements, buffer dilution is usually conducted, such

as in 0.1X PBS or 0.01X PBS, with a Debye length of 2.4 nm and 7.4 nm, respectively. However, diluting the buffer solution changes the ionic strength which may change the structure, resulting in a loss in the aptamer activity and binding affinity [3]. Because the intent was to use the selected aptamers from this work in an electrochemical sensor and an electrolyte-gated field-effect transistor, we had to take the Debye length into consideration and select aptamers with short sequences.

Supporting Note #4. Smart-SELEX for sequence design

After generating the ss-RNA library, it was then filtered. The filtration conditions were chosen based on the literature review and some logical assumptions based on future applications. First, by modeling the secondary structure of each candidate using the Vienna RNA package, and then by considering the sequences with free energy lower than -5 kcal/mol and at least 10 (up to 20) non-hybridized nucleotides. This systematic sorting was done in order to use the aptamers both in the liquid and gas phase:

- Table S1 lists the free energies of the secondary structures of the different aptamers. Notably, all the well-studied aptamers have a free energy greater than -5 Kcal/mol. Accordingly, the first criterion used for filtering the aptamers was that the free energy of the secondary structure must be greater than -5 Kcal/mol.

Table S1. The free energy secondary structure of RNA aptamers binding different ligands.

Ligand	Aptamer length	Aptamer free energy (Kcal/mol)
ATP	40	-17.7
Theophylline	33	-11.6
Isoleucine	27	-7.30
Gentamicin	27	-13.9

- The second criterion was that the aptamers had at least 10 free nucleotides so that the RNA aptamer (as shown in the figure below) will create a pocket that can be used to dock the target inside of it. Moreover, we wanted to ensure that during the interaction between the aptamer and the analyte the electrostatic interactions (van der Waals forces, hydrogen bond) remains dominant. Hence, the more free nucleotides aptamers have, the more electrostatic interactions there will be.

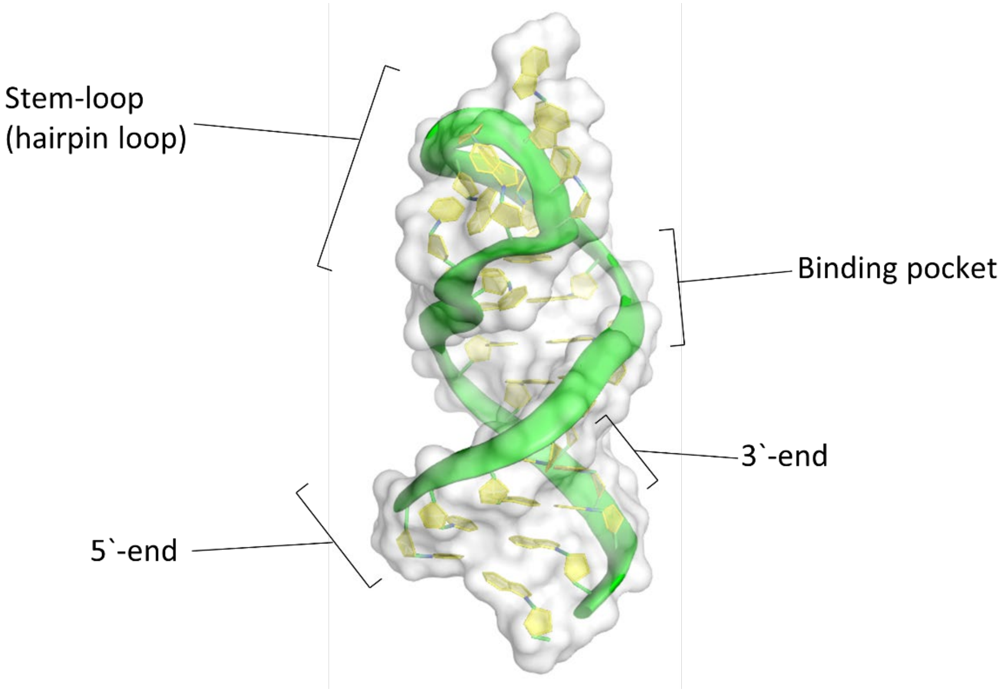

Figure S1. The 3D structure of aptamer1 extracted from molecular dynamic simulation.

Supporting Note #5. Predicting the binding probability using machine learning

In this approach, a Convolution Neural Network model that uses CNN-blocks to learn and extract the features from the aptamer's sequences and targets' physicochemical properties was developed. Hyperparameters were selected after cross-validation using the external unseen validation dataset (shown in Table S1).

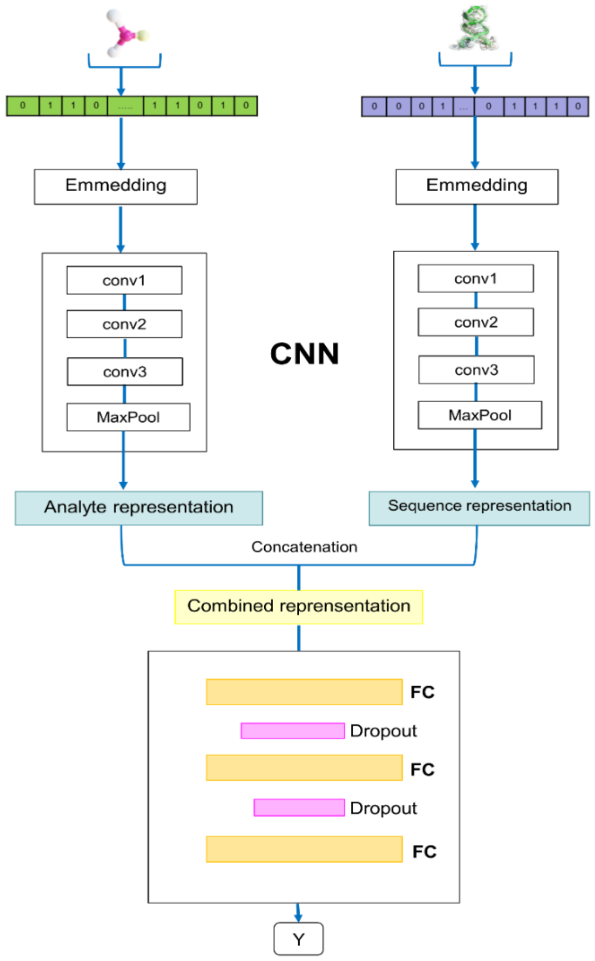

Figure S2. Deep-SELEX model structure.

Table S3. selected values of hyperparameters for Deep-SELEX.

Parameters	Range
Learning rate	0.0001
Batch size	256
Epoch	25
Optimizer	Adam
Hidden neurons	1024; 1024; 512
Number of filters	32; 64, 96
Filter length compounds)	{6, 12, 18}
Filter length (proteins)	{2, 4, 8}
Dropout	0.1

We adopted a model where CNN-blocks were used on all the inputs (both aptamer sequence and target molecules) to learn representations and extract features.

The candidates resulting from the previous stage (filtration step) were fed into the trained CNN model to predict the binding state (yes or no) between the candidate aptamer and the target (NH_4^+). After this stage, the number of candidates was lowered to 38,327, which were then used as an input for the next stage (docking).

Supporting Note #6. Docking

First, the binding energy and the binding state between the positive target (NH_4^+) and the best 3000 candidate aptamers from the previous step were predicted using molecular docking; then, the same was done between the candidates with high affinity towards NH_4^+ and the negative targets to eliminate the candidates with cross-sensitivity. The predicted binding energies of the candidates were in the range of -4.19 to -6.6 Kcal mol^{-1}. At the end of this step, we had 18 candidates with the lowest binding energy towards NH_4^+ and high binding energy towards the negative targets (TMA and DMA). Table S2 shows the best 10 candidates with high affinity towards NH_4^+.

Table S4. Top 10 candidate sequences.

Rank	Candidate sequences
Ap-tamer 1	CCAUGUAAGCGCGGUACU-CUUACGUGA
Ap-tamer 2	UCGCGUCUAGCCCAU-UGAUAGGCCCGA
Ap-tamer 3	UCCACGUGGUG-CCAUACUCCGGCGUGG
Ap-tamer 4	CCUCUCAGGCUUGUACUGCCAC-GAGGA
Ap-tamer 5	GCCCUGGGCCGCUCAUUCCCU-CUGGCU
Ap-tamer 6	ACGGCCAACCUGCGGUCACUG-CACCGG
Ap-tamer 7	GCCUCCGAGACCCUCCCGGCAAC-GGCU
Ap-tamer 8	CCCAUACUAUACCGUACAUGUG-CGGGG
Ap-tamer 9	GUAUUACCACCGGUACGGGA-GAGUGCA

Ap-tamer 10	CCGUCCGGGGCUGAU-UAUAGGCACGGU

Supporting Note #7. Required time

Three of the initial steps were carried out using Python scripts, including selecting RNA candidates, filtering the RNA candidates, and performing Deep-SELEX. These first three steps can be completed very quickly (in a matter of minutes). Docking and molecular dynamic simulations, in contrast, are the most time-consuming steps. For instance, docking was performed on 38,327 RNA candidates (positive docking) and 1896 (negative docking) "TMA and DMA" candidates. To reduce the docking time, a Message Passing Interface (MPI) scheme was implemented. Taking into account the fact that every candidate took approximately 5 seconds, this process took approximately 2 days for positive docking and 6 hours for negative docking. After that, molecular dynamic simulations were conducted on the top five candidates. As each 100 ns simulation took around 1.5 days, the entire process took around 8 days. Therefore, selecting a new aptamer took a total of about 10 days.

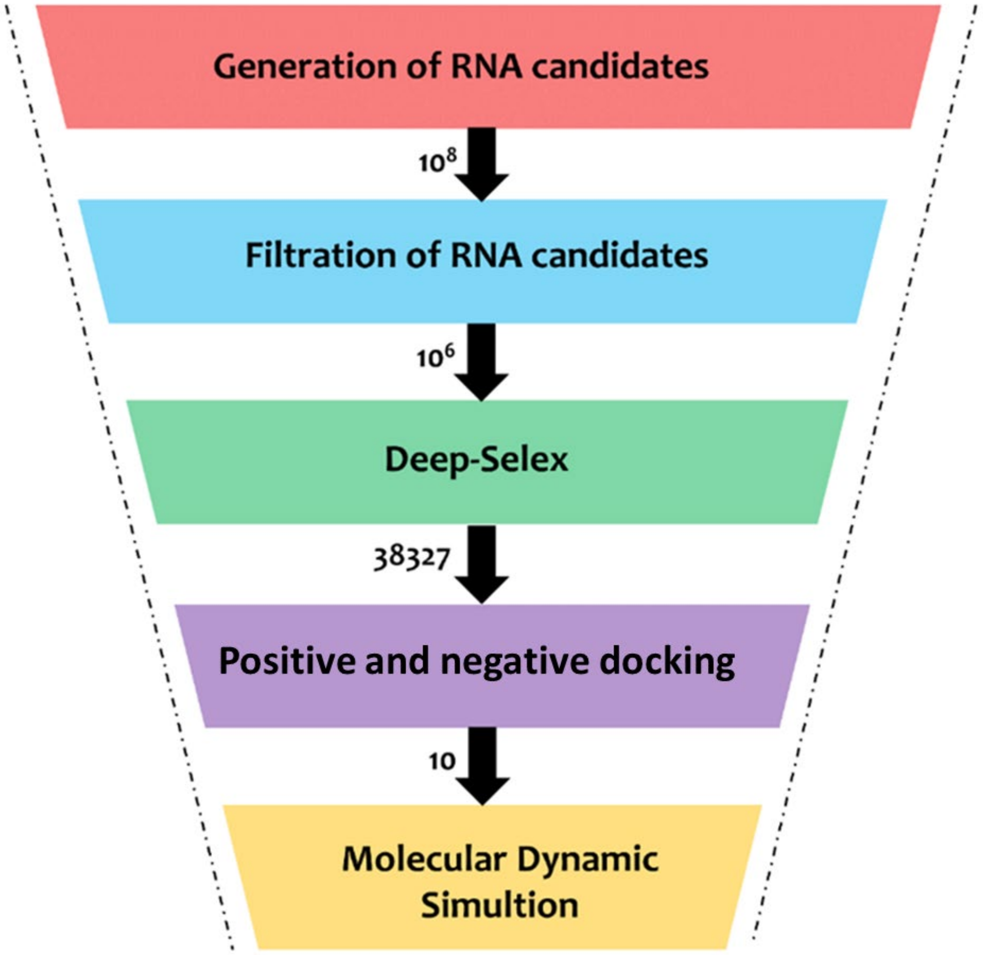

Figure S3. Evolution of the number of RNA candidates through the Smart-SELEX approach.

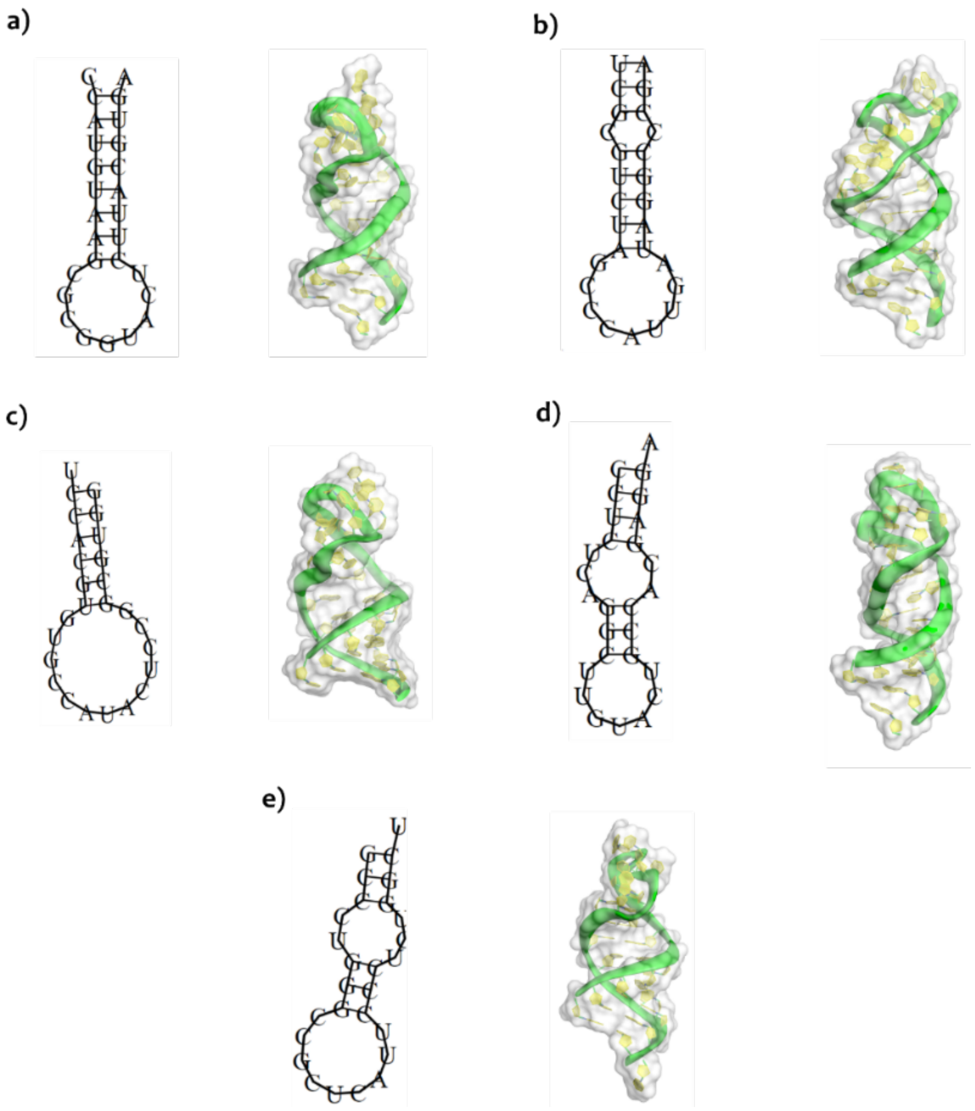

Figure S4. The secondary and tertiary structures of five top aptamers.

Supporting Note #8. Sensor characterization

As reported in Figure S5(a), the IV curve shows a pair of well-defined redox peaks obtained from the bare electrode and after the immobilization of the aptamers. The bare electrode redox peak (I_P = 12.49 mA) is related to the high electron transfer between the electrolyte solution $[Fe\ (CN)_6]^{3-/4-}$ and the electrode surface. This value is higher than the ones reported in the literature (carbon or gold electrodes) due to the high conductivity of the silver ink [4]. The choice of silver as electrode material, allows for enhancing the sensitivity of the aptasensor. The immobilization of the aptamers onto the bare electrode decreased the redox current peak to 8.02±0.95 mA; this change is in agreement with previously reported works [4] [5]. This decrease might be ascribed to the presence of an RNA backbone that is composed of sugars and phosphate groups. The phosphate groups are negatively charged, which makes the aptamer's overall charge strongly negative; hence, the $[Fe\ (CN)_6]^{3-/4-}$ anions are repelled from the electrode surface by the immobilized aptamers which in this case acts as a barrier for the electron transfer [5].

EIS is an electrochemical tool used for monitoring the interfacial change of the electrode surface. Figure S5(b) illustrates typical Nyquist plots obtained before and after the immobilization of the aptamers. The Randles equivalent circuit was used to fit the impedance output and to explain the impedance output, moreover, it was used to relate the biological and the electrical domains [6]. In this model (Figure S5(b) – Inset), C describes the capacitance created between the electrode surface/electrolyte; the Warburg element (Z_w) models the mass diffusion of the anions in the electrolyte (bulk) towards the electrode surface; R_s is the electrolyte resistance, due to the use of the same electrolyte during the EIS experiments the values were almost constant (25–30 Ω); and R_{et} models the electron transfer between the electrode surface and the electrolyte. The latter is represented by the semicircle at low frequencies. The bare electrode had a low R_{et} value (1412 Ω) due to the low electron transfer resistance. Instead, the immobilization of the aptamers on top of the bare electrode increased the R_{et} to 5156 Ω. This may be due to the electrostatic repulsive interaction between the negatively charged phosphate groups of the aptamers and $[Fe(CN)_6]^{3-/4-}$ (used as a supporting electrolyte) anions [7]. Thus, the aptamers increased WE surface resistance and acted as an obstacle to the electron transfer rate, as observed from the CV analysis. Finally, the results of CV and EIS agreed with the Infrared spectra (see SI Figure S4), indicating the successful immobilization of the aptamers.

Figure S6(a) and S6(b) show the infrared (IR) spectrums recorded during aptasensor fabrication, before and after the immobilization of the aptamers, respectively. The spectrum presents the Ag-11-Mua, where the peak at 500 cm^{-1}, corresponds to the Ag-S stretching vibration, confirming the chemical attachment of 11-Mua on the electrode surface [8]. The peak at 1633 cm^{-1} can be assigned to the stretching of the C=C bond, while the peak at 1720 cm^{-1} and 2820 cm^{-1} can correspond to the C=O and C-H stretching vibration of R–COOH. After the immobilization of the aptamers, the IR spectrum (Figure S6(b)) shows the presence of the symmetric deformation of $–CH_3$ at 1354 cm^{-1}, symmetric stretching of C-N at 1398 cm^{-1}, and the disappearance of the C-H peak at 2820 cm^{-1}; moreover, the strong adsorption peak at 1671 cm^{-1} corresponds to the C=O formed at the interface Ag-11-Mua-COOH and aptamer, proving the absorption bands of aptamer on top of the Ag electrode. These results confirmed the successful immobilization of aptamers on top of the Ag working electrode, and, therefore, the attachment of the aptamers on the electrode surface.

It is imperative that in order to optimize the performance of the aptasensor, experimental parameters such as the incubation time and pH needed to be optimized. Hence, the two parameters were adjusted.

- The incubation time of NH4+ is an important parameter that needed to be optimized. Therefore, different incubation times ranging from 5 min to 60 minutes were tested. Finally, it was found that after 15 min of incubation time the aptasensor reached a plateau and 15 min was selected as the incubation time.
- Since the pH of PBS has a crucial effect on the 3D structure of the aptamers, hence the selectivity, it also needed to be optimized in the PBS solution used for preparing the analyte. To find out the optimum pH level of PBS, different pH solutions were tested ranging from 4 to 10. Finally, it was found that pH 7 gave the best performance.

Table S5. The sequences of the aptamers and their modifications.

Aptamer	Candidate sequences	5′-Mod.	3′-Mod.
Aptamer 1	CCAUGUAAGCGCGGUACUCUUAC-GUGA	Aminolink C6	-
Aptamer 2	UCGCGUCUAGCCCAU-UGAUAGGCCCGA	Aminolink C6	-
Aptamer 3	UCCACGUGGUG-CCAUACUCCGGCGUGG	Aminolink C6	-
Aptamer 4	CCUCUCAGGCUUGUACUGCCAC-GAGGA	Aminolink C6	-
Aptamer 5	GCCCUGGGCCGCUCAUUCCCU-CUGGCU	Aminolink C6	-
Control	CUGAAGGCAGUCCGGUG-CCCGGCCGGG	Aminolink C6	-

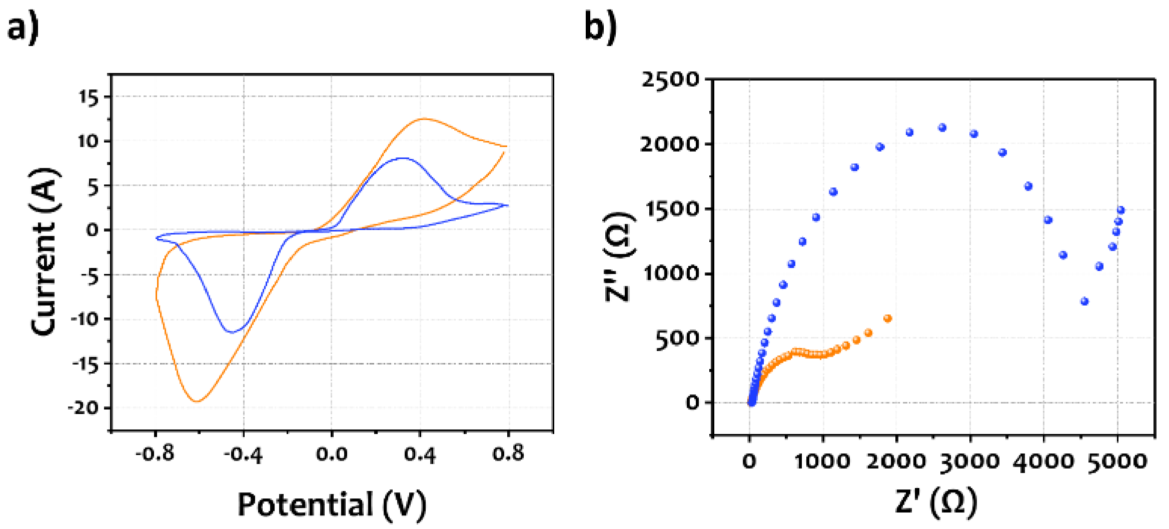

Figure S5. (a) Cyclic voltammogram curves and (b) EIS spectra of the stepwise modified electrode in 1 mM [Fe (CN)6]$^{3/4-}$ an aqueous solution containing 0.1 M KCl: bare (in blue), bare-aptamer (in orange).

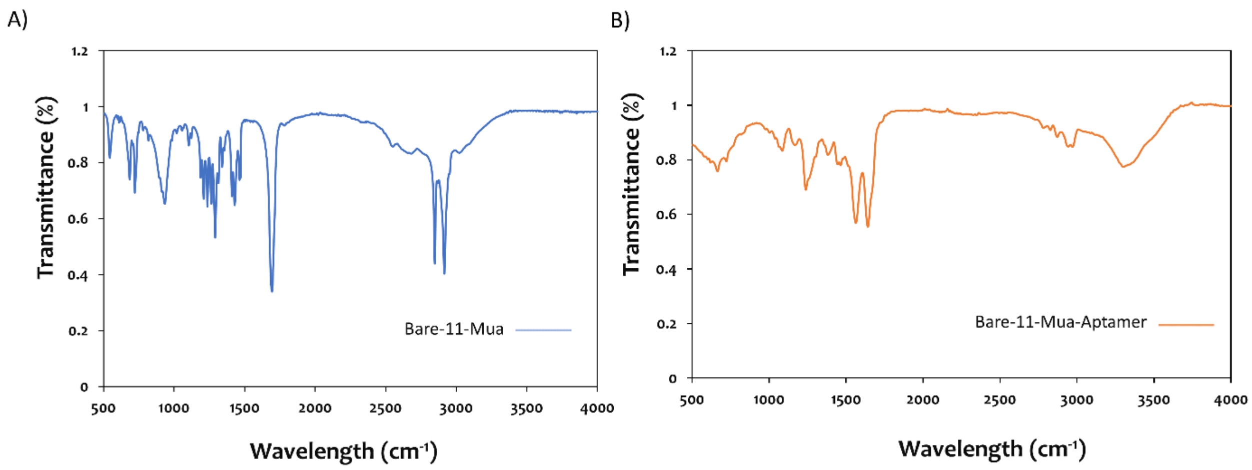

Figure S6. IR spectra of the stepwise modified electrode.

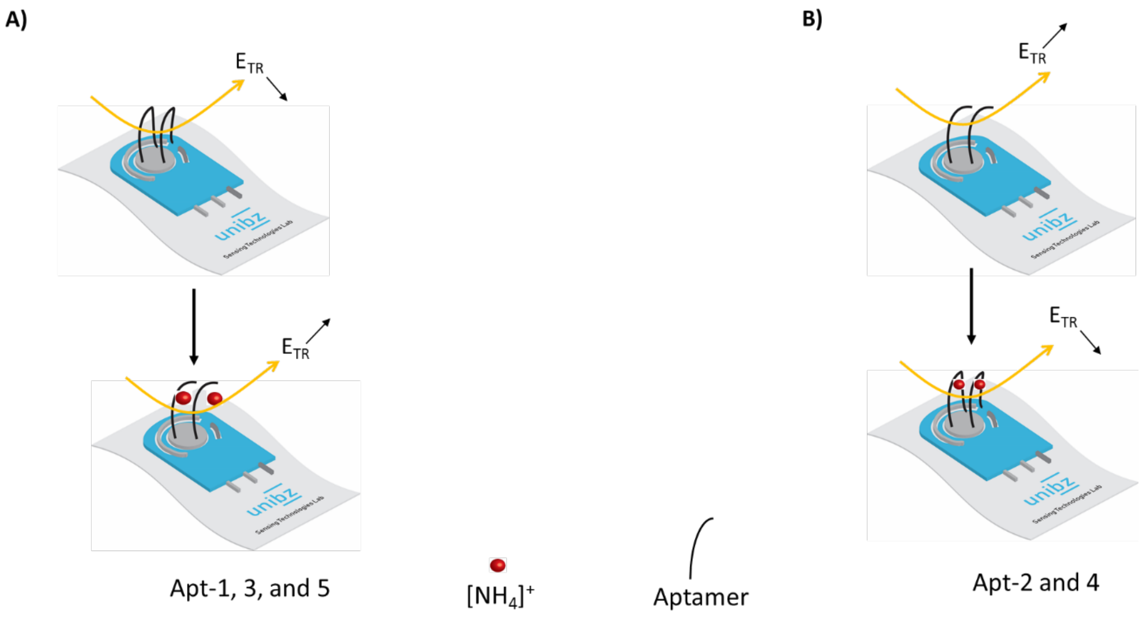

Figure S7. Different conformational changes of the aptamers and the effect on the electron transfer charge (ETR). A) after the interaction with the analyte the aptamers become less compact and bend far from the surface of the working electrode hence, increasing the ETR. B) After the interaction with the analyte, the aptamers undergo a conformational change and bend closer to the surface of the working electrode, hence the ETR decreases.

Supporting Note #9. Molecular Dynamics Simulation

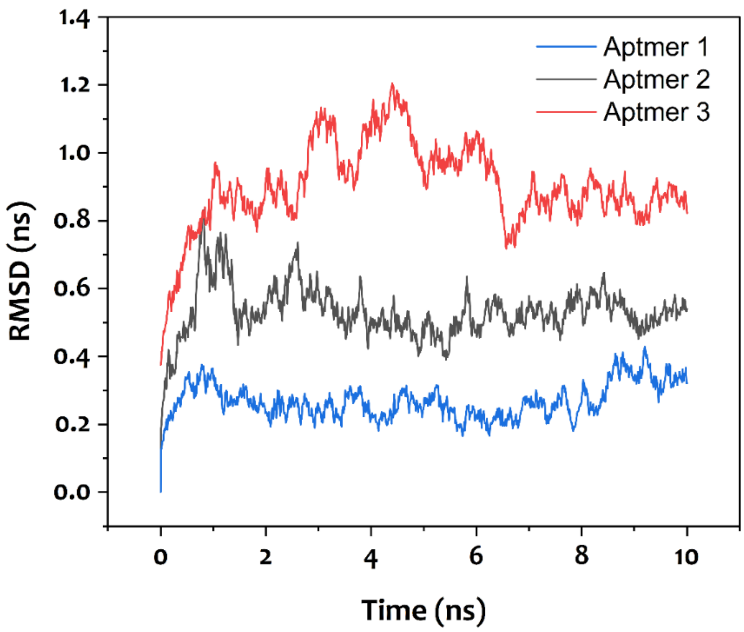

Figure S8. RMSD values of aptamer1, aptamer2, and aptamer3.

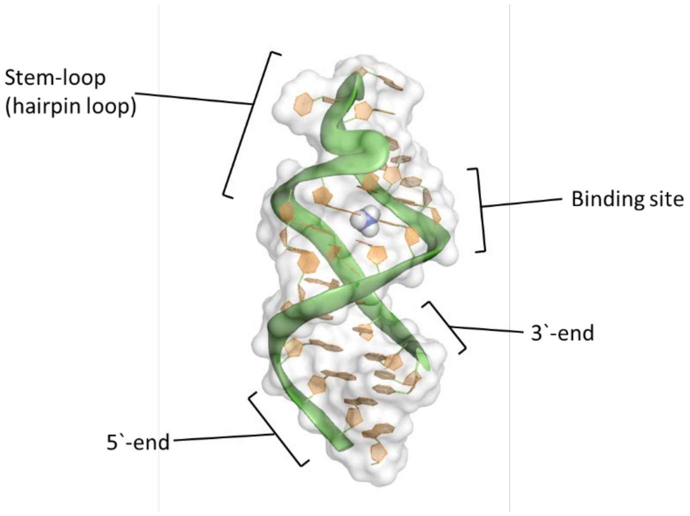

Figure S9. The 3D structure of aptamer1 interacted with ammonia extracted from the molecular dynamic simulation.

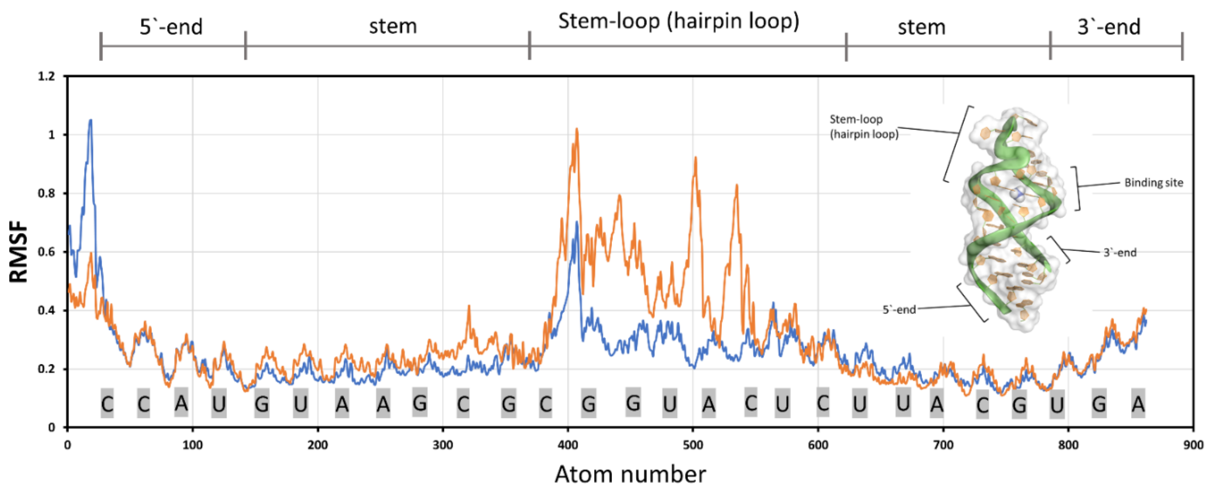

Figure S10. The RMSF of aptamer 1 atoms during 25 ns MD simulation. The RMSF comparison between aptamer (orange line) and Aptamer1+NH_4^+ (blue line).

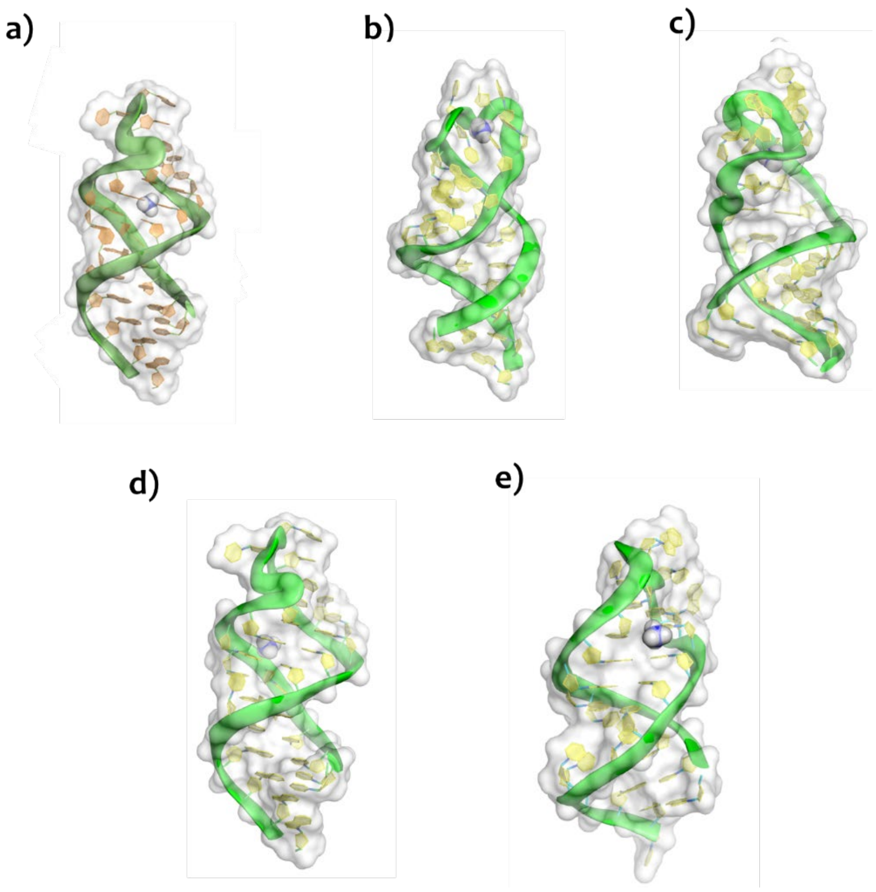

Figure S11. The 3D structure of the five top aptamers interacted with ammonia extracted from molecular dynamic simulation: a) aptamer1, b) aptamer2, c) aptamer3, d) aptamer4, and e) aptamer5.

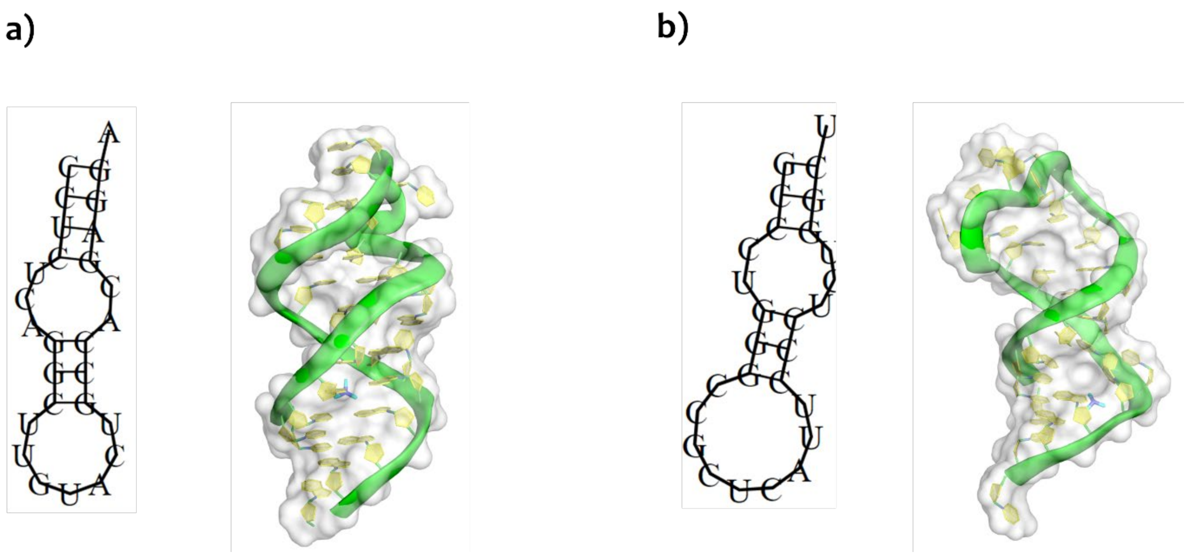

Figure S12. The 3D structure of a) aptamer4 and b) aptamer5 interacted with TMA.

Supporting Note #10. Sensors regeneration and stability

- Sensors regeneration

Regeneration experiments of aptasensor1 were conducted by rinsing the sensor in 5 M urea for 10 minutes. As a result of two regeneration cycles, the sensor response was reduced to 79.13 ± 3.60 %, and after five regeneration cycles, it was reduced to 47.66 ± 2.51 % (Figure S8). The loss in performance may be due to the fact that RNA aptamers are less stable compared to DNA aptamers and/or due to the usage of Urea.

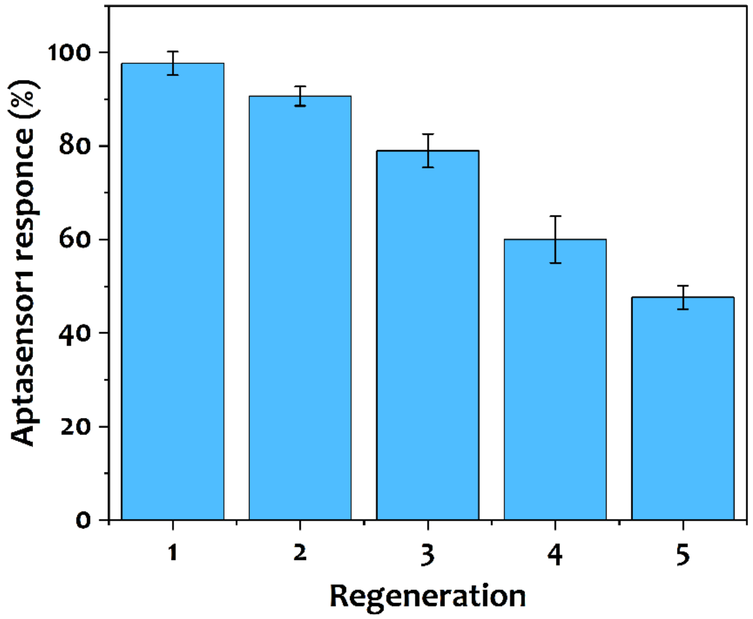

Figure S13. The change in aptasensor1 response after different cycles of regenerations (detecting 100 mM of ammonia).

- Sensor stability:

Furthermore, the stability of the aptasensor1 over time was examined. The sensors were kept at 4 C° for 15 days and the response of three different sensors was examined every five days. After five days, the sensors' response decreased to 94.68 ± 2.08; this good stability may be attributed to the use of a self-assembled monolayer. After 15 days, the sensors' response decreased to 78.64 ± 4.04 (Figure S9).

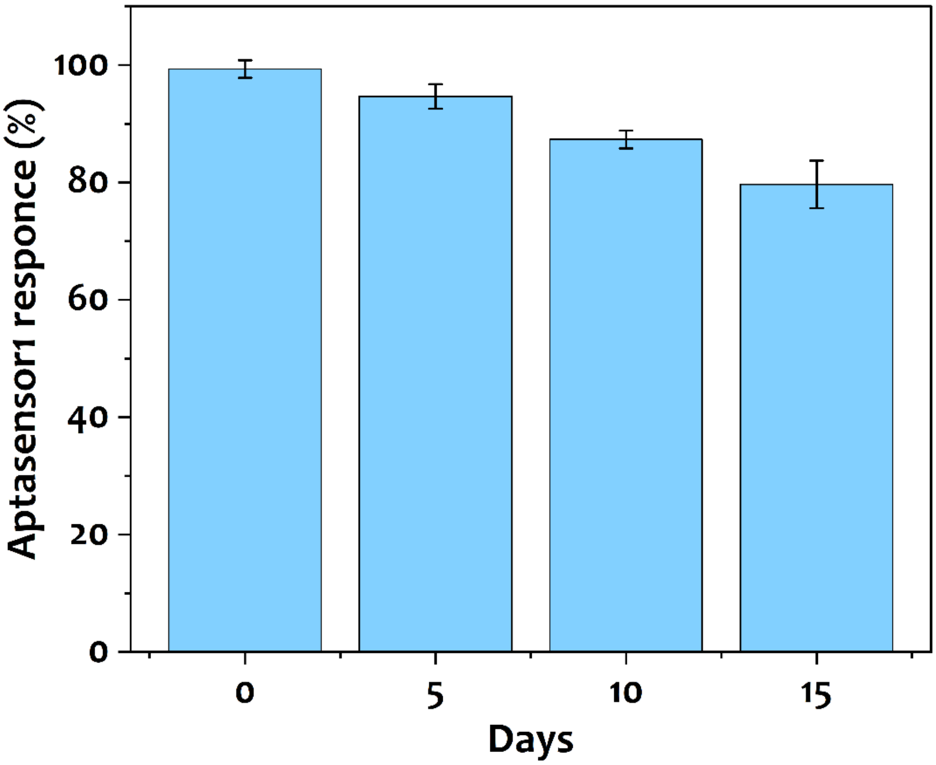

Figure S14. The stability of aptasensor1 over time (detecting 100 mM of ammonia).

Supporting Note #11. Detection of real sample

It is imperative to test the proposed sensor in real water samples to determine its applicability. In this way, the sensor was challenged with a real sample (tap water) using a standard addition procedure. Without any additional preparation or purification, 100 mM of ammonia was added to the real sample. The results are presented in Table 5 with relative recovery (RR). In addition, the results of aptasensor1 were well-concordant with those of the added concentration in tap water, with a RR of 103.46. As a result, the matrix effect was found to be almost negligible for the performance of the proposed aptasensor in real sample analysis.

Table S5. $NH4^+$ detection in a real sample.

Sample	Added (mM)	Detected by sensor (mM)	RR (%)
Tap water	100	103.46 ± 0.84	103.46

References

1. Wang, H.; Wang, Y.; Liu, S.; Yu, J.; Xu, W.; Guo, Y.; Huang, J. An RNA Aptamer-Based Electrochemical Biosensor for Sensitive Detection of Malachite Green. *RSC Advances* **2014**, *4*, 60987–60994.

2. McKeague, M.; McConnell, E.M.; Cruz-Toledo, J.; Bernard, E.D.; Pach, A.; Mastronardi, E.; Zhang, X.; Beking, M.; Francis, T.; Giamberardino, A.; et al. Analysis of In Vitro Aptamer Selection Parameters. *J. Mol. Evol.* **2015**, *81*, 150–161.

3. Nakatsuka, N.; Yang, K.A.; Abendroth, J.M.; Cheung, K.M.; Xu, X.; Yang, H.; Zhao, C.; Zhu, B.; Rim, Y.S.; Yang, Y.; et al. Aptamer-Field-Effect Transistors Overcome Debye Length Limitations for Small-Molecule Sensing. *Science* **2018**, *362*, 319–324.

4. Hassani, S.; Akmal, M.R.; Salek-Maghsoudi, A.; Rahmani, S.; Ganjali, M.R.; Norouzi, P.; Abdollahi, M. Novel Label-Free Electrochemical Aptasensor for Determination of Diazinon Using Gold Nanoparticles-Modified Screen-Printed Gold Electrode. *Biosensors and Bioelectronics* **2018**, *120*, 122–128.

5. Khan, R.; Aissa, S. ben; Sherazi, T.A.; Catanante, G.; Hayat, A.; Marty, J.L. Development of an Impedimetric Aptasensor for Label Free Detection of Patulin in Apple Juice. *Molecules* **2019**, *24*, 1–12.

6. Zhao, Z.; Chen, H.; Ma, L.; Liu, D.; Wang, Z. A Label-Free Electrochemical Impedance Aptasensor for Cylindrospermopsin Detection Based on Thionine-Graphene Nanocomposites. *Analyst* 2015, 140, 5570–5577.

7. Lipfert, J.; Doniach, S.; Das, R.; Herschlag, D. Understanding Nucleic Acid-Ion Interactions. *Annu. Rev. Biochem.* **2014**, *83*, 813–841.

8. Douaki, A.; Abera, B.D.; Cantarella, G.; Shkodra, B.; Mushtaq, A.; Ibba, P.; Inam, A.K.M.S.; Petti, L.; Lugli, P. Flexible Screen Printed Aptasensor for Rapid Detection of Furaneol: A Comparison of CNTs and AgNPs Effect on Aptasensor Performance. *Nanomaterials* **2020**, *10*, 1167.
